# Prenatal brain disruption in isolated sulfite oxidase deficiency

**DOI:** 10.1186/s13023-017-0668-3

**Published:** 2017-06-19

**Authors:** Hsiu-Fen Lee, Ching-Shiang Chi, Chi-Ren Tsai, Hung-Chieh Chen, I-Chun Lee

**Affiliations:** 1 Division of Nursing, Jen-Teh Junior College of Medicine, Nursing and Management, 79-9, Sha-Luen Hu Xi-Zhou Li Hou-Loung Town, Miaoli, Taiwan; 2 Department of Pediatrics, Taichung Veterans General Hospital, 1650, Taiwan Boulevard Sec. 4, Taichung, 40705 Taiwan; 3 School of Medicine, Chung Shan Medical University, 110, Sec. 1, Jianguo N. Rd, Taichung, 40201 Taiwan; 4 Department of Pediatrics, Tungs’ Taichung Metroharbor Hospital, 699, Taiwan Boulevard Sec. 8, Wuchi, Taichung, 435 Taiwan; 5 Institute of Molecular Biology, National Chung Hsing University, 250, Kuo Kuang Rd, Taichung, 402 Taiwan; 6 Department of Radiology, Taichung Veterans General Hospital, 1650, Taiwan Boulevard Sec. 4, Taichung, 40705 Taiwan; 7 Department of Pediatrics, Taichung Tzu Chi Hospital, 88, Sec. 1, Fengxing Rd, Tanzi Dist, Taichung, 427 Taiwan

**Keywords:** Brain disruption, Brain MRI, Isolated sulfite oxidase deficiency, Prenatal period

## Abstract

**Background:**

Isolated sulfite oxidase deficiency (ISOD) is a very rare autosomal recessive inherited neurometabolic disease. The most striking postnatal neuroimaging finding is multicystic encephalomalacia, which occurs rapidly within days to weeks after birth and mimics severe hypoxic-ischemic encephalopathy. The aim of this study was to describe the prenatal neuroimaging features in a neonate and a fetus diagnosed with ISOD.

**Results:**

We report an 11-day-old female neonate who presented with feeding difficulties, decreased activity, neonatal seizures, and movement disorders within a few days after birth. Brain MRI at 9 days of age showed cystic lesions over the left frontal and temporal areas, diffuse and evident T2 high signal intensity of bilateral cerebral cortex, and increased T2 signal intensity of the globus pallidi. A pronounced low level of plasma cysteine and normal level of plasma uric acid were noted. Mutation analysis of SUOX revealed homozygous c.1200C > G mutations, resulting in an amino acid substitution of tyrosine to a stop codon (Y400X). The diagnosis of ISOD was made. The brain MRI of a prenatally diagnosed ISOD fetus of the second pregnancy of the mother of the index case showed poor gyration and differentiation of cortical layers without formation of cystic lesions at gestational age 21 weeks.

**Conclusion:**

Cystic brain destruction might occur prenatally and neurodevelopment of gyration and differentiation of the cortical layers in the developing brain could be affected by sulfite accumulation early during the second trimester in ISOD patients. This is the first description of the prenatal neurodevelopment of brain disruption in ISOD.

## Background

Sulfite oxidase (SO), a molybdenum-containing enzyme located in the intermembrane space of the mitochondria, catalyzes the oxidation of sulfite to sulfate, the final step in the degradation of sulfur-containing amino acids and environmental sulfites, and thus human cells are protected from its toxic effects. SO deficiency, caused by isolated sulfite oxidase deficiency (ISOD) or molybdenum cofactor deficiency (MoCoD), is biochemically characterized by the accumulation of sulfite, thiosulfate, and S-sulfocysteine in the tissues and biological fluids of the affected patients [1].

ISOD is a very rare and devastating autosomal recessive inherited neurometabolic disease caused by mutations in the sulfite oxidase gene (SUOX). The gene response for sulfite oxidase (SUOX; OMIM 606887) maps to chromosome 12q13.2. Patients with neonatal onset ISOD generally present with a severe and often fatal disease course during infantile and neonatal stages. The main clinical symptoms at disease onset include feeding difficulties, irritable crying, neonatal seizures, profound developmental delay, abnormal muscle tone, and abnormal movements such as choreoathetosis and dystonia. Ectopia lentis may be detected later [2]. The biochemical abnormalities typically reported include excess urinary excretion of sulfites and S-sulfocysteine, with low plasma cysteine [3]. As we reported previously, the striking neuroimaging findings are diffuse cortical swelling at disease onset followed by rapid evolution to multicystic encephalomalacia over the bilateral cerebral cortices and signal changes over bilateral basal ganglia and thalami within days to weeks, which mimics hypoxic-ischemic encephalopathy [4]. Other neuroradiologic findings include hypoplasia of the corpus callosum and multiple older cerebral hemisphere infarction with the features of diffuse cystic degeneration of the supratentorial white matter, along with signal changes over the bilateral basal ganglia and thalami, which could be detected as early as a couple of days after birth [5].

Prenatal brain disruption in an ISOD patient has been reported [6]. Here we report an index case diagnosed with ISOD by a characteristic neuroimaging finding of multicystic encephalomalacia found shortly after the baby was born, a significantly low level of plasma cysteine, and SUOX gene mutations. Brain magnetic resonance imaging (MRI) findings in a prenatally diagnosed ISOD fetus, the second pregnancy of the mother of the index case, were noted.

## Methods

The index case, the first daughter of non-consanguineous parents from Taiwan, was born after an uneventful pregnancy and normal spontaneous delivery at gestational age of 40 weeks. Her birth weight, height, and head circumference were all between the 15th and 50th percentile. The family history was unremarkable.

**Fig. 1:**
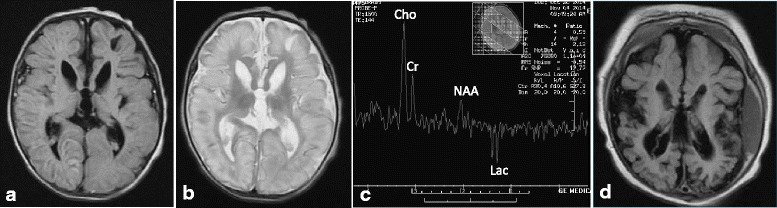
The initial and follow-up brain MRIs of our index case. **a**-**c** The initial brain MRI at age of 9 days. Axial images, FLAIR image (TE 110 ms, TR 8802 ms) (**a**) and T2-weighted image (TE 100 ms, TR 6000 ms) (**b**), show cystic lesions over the left frontal and temporal areas, diffuse and obvious high signal intensity over bilateral cerebral cortex, and signal change over the bilateral globus pallidi. Single-voxel proton magnetic resonance spectroscopy of the cortical gray matter (**c**) reveals elevated Choline (Cho) and abnormally low N-acetylaspartate (NAA) peaks, together with a large lactate (Lac) doublet at intermediate echo time (TE 144 ms). **d**. The follow-up brain MRI at age of 4 months. Axial FLAIR image (TE 122 ms, TR 9002 ms) shows ventricular dilatation, dramatic cerebral cortical atrophy, multicystic lesions over bilateral occipital areas, and subdural hemorrhage

The girl presented with feeding difficulty characterized by poor sucking power and prolonged feeding time at the first day of life. The symptoms worsened and she exhibited decreased activity at the 5th day of life. She was admitted to a neonatal unit. During the hospitalization, subtle seizures with clinical feature of bicycling of legs, alternating myoclonic seizures with rhythmic jerking over limbs, and obvious high-pitched irritable crying developed at the 8th day of life. Brain MRI at the 9th day of life revealed ventricular dilatation, cystic lesions over the left frontal and temporal areas, diffuse and evident T2 high signal intensity of the bilateral cerebral cortex, and increased T2 signal intensity of the globus pallidi (Fig. 1a and b), as well as an inverted lactate doublet on the proton magnetic resonance spectroscopy (MRS) (Fig. 1c). She was transferred to our hospital for further evaluation.

Upon admission, the neurological examinations revealed poor eye contact, intact cranial nerves except for poor sucking and swallowing power, brisk deep tendon reflexes with extensor plantar reflex, a positive ankle clonus, generalized hypertonicity, rigidity, and intermittent dystonic posture. Electroencephalography revealed diffuse low amplitude in background activity. Metabolic workups revealed normal bicarbonate and ammonia levels, and a normal level of plasma uric acid 5.6 mg/dl (normal reference 2.4-7.2). Assays of tandem mass spectroscopy, including profiles of amino acids and acylcarnitine, and urinary organic acids were within normal limits. Urine sulfite strip test did not detect the presence of sulfites. However, a very low level of plasma cysteine 3.72 umol/L (normal reference 39–191) was detected. Taken together, multicystic encephalomalacia, significant hypocystinemia, and a normal level of plasma uric acid suggested a diagnosis of ISOD. After obtaining informed consent from the patient’s parents, SUOX genetic analysis was conducted.

## Results

### Index case

**Fig. 2:**
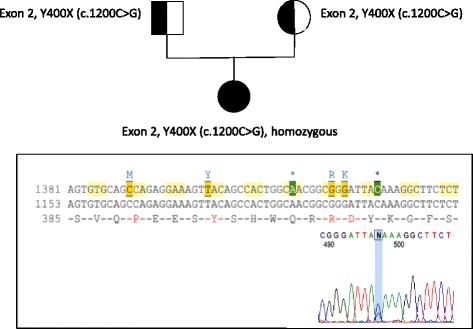
SUOX gene analysis of our index case. Mutation analysis of SUOX revealed homozygous c.1200C > G mutations, resulting in an amino acid substitution of tyrosine to a stop codon (Y400X)

Mutation analysis of SUOX revealed homozygous c.1200C > G mutations, resulting in an amino acid substitution of tyrosine to a stop codon (Y400X) (Fig. 2).

At the age of 4 months, the follow-up brain MRI showed apparent cerebral cortical atrophy, multiple and small cystic lesions over bilateral occipital areas, and subdural hemorrhage over the left frontal and temporal areas (Fig. 1d). The patient required enteral feeding which proceeded to gastrostomy at the age of 5 months. Her last eye fundus examination at the age of 2 years 3 months showed a normal finding without any indication of ectopia lens. At the time of writing, she was 2 years 4 months old and bedbound with rigid limbs, intermittent, evident dystonic posture along with screaming episodes, and no eye contact. Myoclonic seizures and multifocal seizures persisted and were refractory despite multiple antiepileptic drugs administration. Dysmorphic face with microcephalus and mild forehead wrinkles was noted.

### The ISOD fetus, the second pregnancy of the mother of the index case

**Fig. 3:**
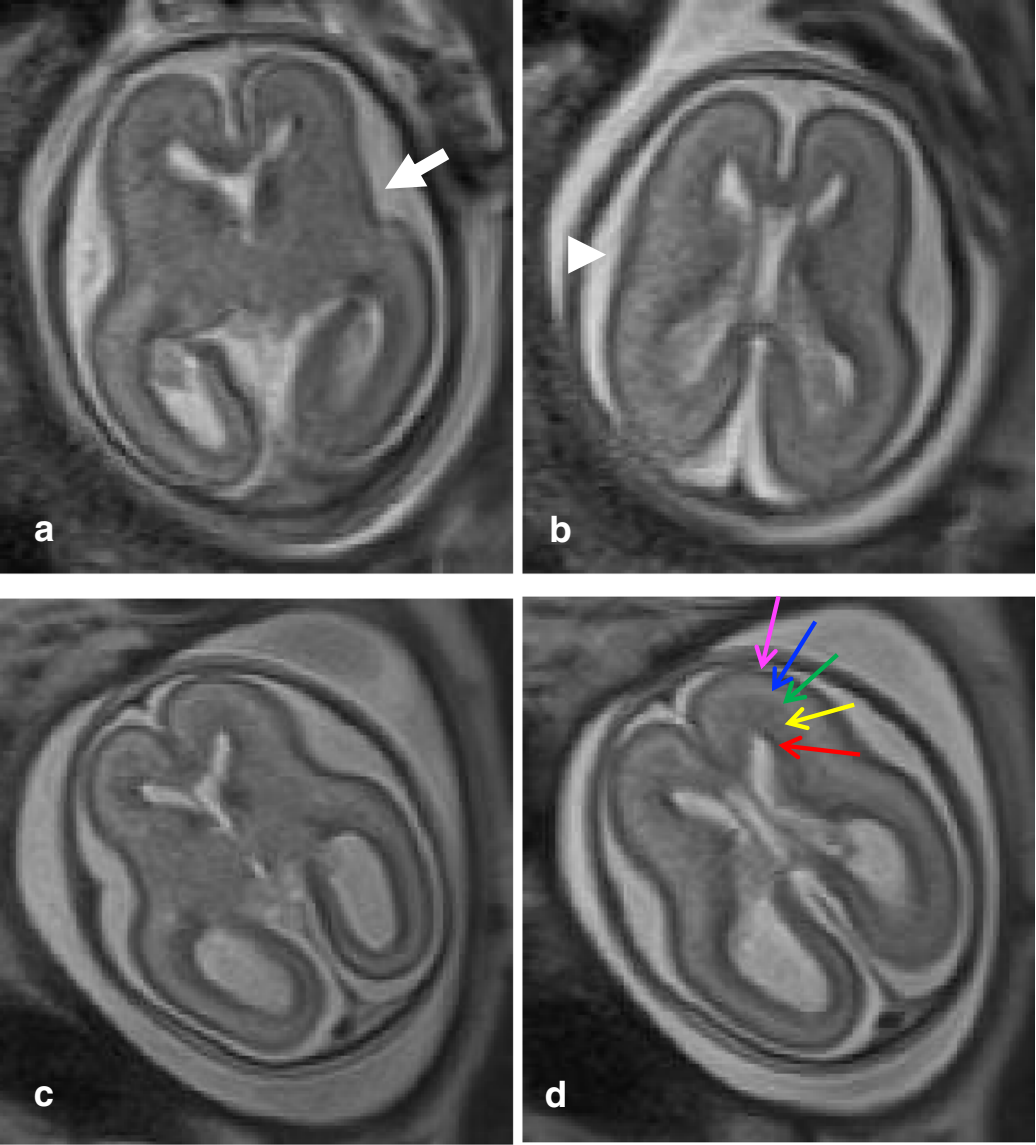
Brain MRI of the ISOD fetus and the normal control images of brain development at gestational age 21 weeks. **a** and **b**, Axial brain images (TE 93 ms, TR 1400 ms, Matrix 256 × 166, FOV 200 nm, Slice 3 mm, Voxel size 0.98 × 0.78 × 3 mm^3^) of the ISOD fetus at gestational age 21 weeks show poor gyration at left sylvian fissure (arrow) (**a**) and right lateral sulcus (arrow head), and poor differentiation of the cortical layers (**a** and **b**). **c** and **d**, Axial brain images (TE 96 ms, TR 1400 ms, Matrix 256 × 166, FOV 200 nm, Slice 3 mm, Voxel size 0.98 × 0.78 × 3 mm^3^) of the normal control at gestational age 21 weeks show normal gyration at the sylvian fissure (**c**) and lateral sulcus (**d**). Normal 5 layered appearance of the brain cortex, from medial to lateral, shows ventricular zone/ germinal matrix (red arrow), periventricular zone (yellow arrow), intermediate zone (green arrow), subplate (blue arrow), and cortical zone (pink arrow) (**d**). This finding indicates that neurodevelopment is affected early in the prenatal stage

The mother of the index case had an unplanned pregnancy after 1 year of the index case being born. Prenatal SUOX genetic analysis from amniocentesis at gestational age 17 weeks revealed homozygous c.1200C > G (Y400X) mutations. Before termination of the pregnancy, the mother agreed to perform a brain MRI to investigate the brain of the fetus. At gestational age 21 weeks, the brain MRI of the fetus showed poor gyration and differentiation of cortical layers without formation of cystic lesions (Fig. 3). This is the first description of prenatal disruption of neurodevelopment in a fetus with ISOD.

## Discussion

The neuroimaging findings of the index case showed more mature cystic lesions over the cerebral hemisphere coexisting with fulminant injury to both the gray and white matter structure of the bilateral cerebral hemispheres that occurred in the days after the baby was born. Prenatal multicystic encephalomalacia in an ISOD patient [6] and prenatal brain disruption in a MoCoD-related SO deficient patient [7] were demonstrated by brain sonography, which showed the replacement of subcortical white matter by progressive multiple cystic lesions at the third trimester. We speculate that brain destruction caused by more mature cystic lesions in the index case developed during the prenatal stage. In addition, analysis of the neuroimaging study of the ISOD fetus revealed the neurodevelopment process of gyration and differentiation of the cortical layers was affected early in the second trimester. Based on the evidence from neuroimaging findings in our cases, we concluded that the neurodevelopment disruption and brain destruction in ISOD patients occur prenatally. Serial investigations of the developing brain in utero with brain ultrasonography or MRI would help to gain a better understanding of the exact timing of sulfite toxicity. Such highly relevant data would be invaluable in the evaluation of the timing and risks/benefits of early delivery and interventions.

ISOD is biochemically characterized by tissue accumulation and high urinary excretion of sulfite, thiosulfate, and S-sulfocysteine. Although the neuroimaging findings of ISOD patients revealed that brain destruction occurred prenatally, the majority showed normal growth of head circumference and no prenatal abnormality during the antenatal examination. One possible interpretation of this time course is that the maternal-placental circulation may partially regulate the clearance of sulfite level in utero until the cord is clamped, which isolates the neonatal circulation leading to a greatly reduced transfer of SO from the mother to the baby [8]. Sulfite accumulation in the brain and tissue fluids increases, resulting in the rapid onset and rapid deterioration of clinical symptoms, along with a dramatic acceleration in the destruction of subcortical white matter and gray matter, as evidenced by the clinical presentations as well as the diffuse and obvious signal changes of bilateral cerebral cortices in the index case.

The neuropathological findings of ISOD patients include massive neuronal loss and gliosis in the cerebral cortex, atrophy of the cerebral white matter with small scattered cysts, marked atrophy of the basal ganglion, and evident myelin loss in the cerebellum [2]. Experimental studies have proposed mechanisms underlying the neuropathology of this disorder which involve the disruption of brain mitochondrial energy and redox homeostasis by the excess amount of sulfite and thiosulfate, leading to the impairment of the electron flow through the respiratory chain. Furthermore, the excess sulfite acts synergistically with Ca^2+^ to induce a change in mitochondrial permeability. As a result of these biochemical reactions, the brain fails to produce sufficient energy. The consequences of bioenergetic and redox homeostasis dysfunction may contribute to the neurological damage found in these patients [9, 10]. Clinically, accumulation of high amounts of lactic acid in some affected patients indicated mitochondrial dysfunction [11]. Data from MRI and proton MRS also suggested an energy deficit in human ISOD related to dysfunction of brain mitochondria [12], which elevated the concentration of lactate in vivo, as demonstrated in the index case.

## Conclusion

ISOD should be considered in all neonates and infants with early formation of multicystic encephalomalacia but no obvious insults. With respect to diagnostic clues, hypocystinemia and normal level of uric acid are characteristic biochemical findings of ISOD. Cystic brain destruction might occur prenatally and neurodevelopment of gyration and differentiation of the cortical layers in the developing brain could be affected by sulfite accumulation early in the second trimester. Although ISOD is not treatable, establishing a diagnosis allows for future prenatal testing by mutation analysis of the SUOX gene.

## Abbreviations

ISOD: Isolated sulfite oxidase deficiency
MoCoD: Molybdenum cofactor deficiency
MRI: Magnetic resonance imaging
MRS: Magnetic resonance spectroscopy
SO: Sulfite oxidase
